# High-rate, High Temperature Acetotrophic Methanogenesis Governed by a Three Population Consortium in Anaerobic Bioreactors

**DOI:** 10.1371/journal.pone.0159760

**Published:** 2016-08-04

**Authors:** Dang Ho, Paul Jensen, Maria-Luisa Gutierrez-Zamora, Sabrina Beckmann, Mike Manefield, Damien Batstone

**Affiliations:** 1 Advanced Water Management Centre, The University of Queensland, St Lucia, Queensland, Australia; 2 Centre for Marine BioInnovation, School of Biotechnology and Biomolecular Sciences, The University of New South Wales, Kensington, New South Wales, Australia; Chengdu Institute of Biology, CHINA

## Abstract

A combination of acetate oxidation and acetoclastic methanogenesis has been previously identified to enable high-rate methanogenesis at high temperatures (55 to 65°C), but this capability had not been linked to any key organisms. This study combined RNA–stable isotope probing on ^13^C-labelled acetate and 16S amplicon sequencing to identify the active micro-organisms involved in high-rate methanogenesis. Active biomass was harvested from three bench-scale thermophilic bioreactors treating waste activated sludge at 55, 60 and 65°C, and fed with ^13^-C labelled and ^12^C-unlabelled acetate. Acetate uptake and cumulative methane production were determined and kinetic parameters were estimated using model-based analysis. Pyrosequencing performed on ^13^C- enriched samples indicated that organisms accumulating labelled carbon were *Coprothermobacter* (all temperatures between 55 and 65°C), acetoclastic *Methanosarcina* (55 to 60°C) and hydrogenotrophic *Methanothermobacter* (60 to 65°C). The increased relative abundance of *Coprothermobacter* with increased temperature corresponding with a shift to syntrophic acetate oxidation identified this as a potentially key oxidiser. *Methanosarcina* likely acts as both a hydrogen utilising and acetoclastic methanogen at 55°C, and is replaced by *Methanothermobacter* as a hydrogen utiliser at higher temperatures.

## Introduction

In the anaerobic digestion (AD) process, complete degradation of complex organic matter to biogas (CH_4_, CO_2_) is a result of the sequential and coordinated metabolic activities of different groups of micro-organisms categorised as hydrolytic/fermentative bacteria, acetogenic bacteria and methanogenic archaea [1, 2]. These groups of micro-organisms differ widely in terms of nutrient needs, growth kinetics and sensitivity to environmental conditions; thus maintaining a balance between these trophic groups is challenging yet critical to the success of the process operation. Among the many operational parameters, temperature and solids retention time (SRT) have the most significant influence on microbial communities [3–5].

Methanogens perform the final step in converting biomass to methane and are often regarded as the slowest growing populations within AD. Under mesophilic conditions (30 to 40°C), a minimum retention time of 5 days is required [6, 7] to reduce washout of key trophic groups and maintain balance between the faster-growing acid-producing bacteria and the slower-growing acid-consuming groups. Increasing process temperature from 50 to 70°C has been demonstrated to enhance both kinetic and metabolic activities [8, 9], and has therefore been employed as a strategy to improve process rates when treating sewage sludge and cellulosic waste [5, 10, 11]. Operation at a shorter SRT (< 4 days) means more waste could be treated, or a smaller reactor built, generating more biogas while utilizing the same facility, deferring capital expense, or reducing capital for new sites.

Temperature is also recognised as an important factor regulating methanogenic community structure and biochemical conversion pathways. Acetate is the main source of methane during anaerobic digestion in engineered processes, and the conversion proceeds via two main pathways, namely acetoclastic methanogenesis (AM), and syntrophic acetate oxidation (SAO) followed by hydrogenotrophic methanogenesis (HM). SAO is endergonic (ΔG^o’^ = +104.6 kJ mol^-1^) and strongly influenced by hydrogen concentration. At standard ambient conditions (25°C and 101.3 kPa), acetate degradation is expected to proceed mainly through AM (ΔG^o’^ = -31 kJ mol^-1^) mediated by *Methanosaeta* (also known as *Methanothrix)* or *Methanosarcina* [9]. At elevated temperature (>50°C), SAO becomes energetically more favourable and the association of SAO-HM has been established as an important pathway in several high temperature AD [12–14]. It was recently observed that SAO/AM can occur simultaneously at 55°C [15] and enable high-rate methanogenesis. This particularly indicates there is a balance between the two pathways, especially in cases where they are competing, rather than AM simply being excluded by inhibition (e.g. due to high ammonia concentrations).

As the significance of SAO-HM pathway has now been established, particularly for high-rate and high temperature AD, understanding of mechanisms driving this pathway and active microbes involved in SAO is required. Cultivation and isolation of SAO organisms is extremely difficult. To date, only a limited range of syntrophic acetate oxidising bacteria have been isolated and characterised, including thermophilic bacteria *Thermacetogenium phaeum* [16], *Thermotoga lettinga* [17], and three mesophilic bacteria, *Clostridium ultunense*, *Syntrophaceticus schinkii*, and *Tepidanaerobacter acetatoxydans* [18–20]. Identifying the syntrophic partnership of SAO-HM can be challenging; this is particularly the case in a competitive-interactive environment where (for example) an acetoclastic methanogens can also potentially act as a hydrogenotrophic partner for SAO-HM as seen in Ho et al. [15, 21]. *In situ* techniques such as RNA stable isotope probing (SIP) can identify specifically active members based on the incorporation of ^13^C into the RNA of cells consuming labelled substrates [22–24]. This study uses ^13^C-labelled acetate together with RNA-SIP to identify the active micro-organisms that assimilate labelled substrate at different temperatures.

## Materials and Methods

The overall approach involved growth of stable cultures (fed with waste activated sludge) at 55°C, 60°C, and 65°C in parallel continuous reactors, followed by batch growth in experimental (^13^C-labelled) and control (unlabelled) flasks. The continuous parent reactors were sequenced by 16S-rRNA gene sequencing while the batch experiments were sampled in the growth phase, fractionated by RNA-SIP, and heavy and light fractions from labelled and control experiments sequenced for comparison.

### Anaerobic sludge

Anaerobic sludge was collected from three lab-scale continuous bioreactors (1 L working volume each) treating waste activated sludge collected from a biological nutrient removal process at Elanora Wastewater Treatment Plant, Gold Coast, Australia (-28.117673, 153.443760). Authority to sample was provided by Gold Coast City Council. The bioreactors were operated in parallel at 3 days SRT, which was the minimum SRT required to prevent washout of methanogens as identified in Ho et al. [21], and incubated at 55°C, 60°C, and 65°C, respectively for a period up to 7 weeks. As the bioreactors were continuously stirred the SRT, representing the average retention time of sludge in the reactor, equalled the hydraulic retention time (HRT). Biomass was harvested from each bioreactor at Day 45 after they all reached steady state (defined as stable gas production and low organic acids production, see S1 and S2 Figs).

### DNA extraction

Prior to the RNA-SIP experiment, 5 ml of sludge samples were removed and stored at -20°C for molecular analysis as described in Ho et al. [21]. DNA extractions were performed using FastDNA Spin kits for Soil (MP Biomedicals, Australia) according to the manufacturer’s instructions. The concentrations of each eluted DNA were measured using a NanoDrop ND-1000 spectrophotometer (NanoDrop Technology, Rockland, DE). The extracted DNA was loaded on a 1% agarose gel to identify extent of DNA degradation. Genomic DNA was then subjected to 16S amplicon sequencing which is described below.

### Incubation of sludge samples for RNA stable isotope labelling

Isotopic labelling experiments were conducted in triplicate in 160-ml non-stirred glass serum bottles, followed the method described in Ho et al. [15]. Stock solutions of [^12^C]-unlabelled and [2-^13^C]-labelled acetate (99 atom % ^13^C, Sigma Aldrich) were prepared at 10 g/l in milliQ water. Approximately 3 ml of stock solution was added to 10 ml of fresh biomass (1.8 to 2.0% volatile solids), and combined with basic anaerobic (BA) medium [25] to a final volume of 100 ml. Endogenous methanogenesis was determined in the blank without substrate addition. The serum bottles were immediately flushed with nitrogen, then sealed with butyl rubber stoppers and aluminium crimp seals, and stored in temperature controlled incubators (±1°C). Three sets of experiments were conducted in parallel at 55°C, 60°C and 65°C, using anaerobic sludge collected from continuous digesters at the same temperatures.

Biogas volume and composition were measured daily using a gas chromatograph (GC, Perkin Elmer, USA) equipped with thermal conductivity detector as described elsewhere [15]. Following each biogas measurement, a 1 ml liquid sample was removed to determine residual acetate concentrations using a Perkin Elmer GC with a free fatty acid phase (FFAP) capillary column (Agilent Technologies, USA). The specific methanogenesis activity was then calculated from the linear methane production rate or acetate depletion rate (measured as g COD L^-1^ d^-1^) divided by the biomass VS content (measured as g VS L^-1^) in each series and is presented as the average of the triplicates. Monod kinetics was applied as the dynamic process model for acetate-conversing methanogenesis, shown as:

$$r=-k_{m}\;\frac{S}{K_{S}+S}$$

Where r is the reaction rate (d^-1^), k_m_ is the maximum specific uptake rate (gCOD gCOD_added_^-1^ d^-1^), S is the substrate concentration (gCOD L^-1^), and K_S_ is the half-saturation constant (gCOD L^-1^). The model was implemented in Aquasim 2.1d [26] with parameter uncertainty determined from 95% confidence intervals based on a two-tailed *t-*test based on parameter standard error calculated from the Fisher Information Matrix. A student *t*-test was used to test for significant differences in maximum uptake rate between ^13^C-labelled acetate versus ^12^C-acetate (control) tests (*p* = 0.05 significance threshold) using central slope as an indicator of maximum uptake rate. Normality and comparability of variance was checked in residuals, noting that minor non-normality is corrected in parameter values through the Central-Limit theorem. Residuals were normal but autocorrelated (no correction applied, at p>>0.05).

From the batch activity tests, 10-ml biomass aliquots were removed from the serum bottles on Day 2 for 55°C series, Day 6 for 60°C series, and Day 8 for 65°C series. These sample times were based at the end of maximum uptake when label incorporation would be maximised. Biomass aliquots were immediately centrifuged at 5,000 x *g* for 3 min to remove the supernatant and preserved in 3 volumes of Lifeguard™ soil preservation solution (Mo Bio, USA), and stored at -80°C prior to RNA extraction. All tests were carried out in triplicate, and error bars indicate 95% confidence in the average of the triplicate (two-tailed *t*-test).

### RNA extraction and isopycnic centrifugation

RNA was extracted as previously described [22] using phenol/chloroform and bead beating treatment to enhance cell lysis. Following this, the RNeasy extraction kit (Qiagen, Germany) was used as per the manufacturer’s instructions including the DNase digestion step to remove contaminating DNA. RNA was eluted in nuclease-free water and quantified using the Quant-it-Ribogreen RNA kit (Life Technologies, USA).

^13^C-labelled RNA was then resolved from unlabelled RNA by isopycnic centrifugation following the protocol of Whiteley et al. [27]. Briefly, RNA samples were prepared for density gradient ultracentrifugation by mixing approximately 500 ng of total RNA with 4.24 ml of 1.99 g/ml Cesium trifluoroacetate (CsTFA) solution (GE Healthcare), 181 μl deionised formamide (Sigma Aldrich) and molecular biology grade (MBG) water (Thermo Scientific) to a total volume of 5.3 ml. Gradients were then transferred to 5.1 ml Optiseal polyallomer tubes (Beckman Coulter, NSW, Australia) which were centrifuged in a vertical rotor (P100VT Hitachi) in a Hitachi CP100WX ultracentrifuge (Beckman Coulter, Australia) at 130,000 x *g* for 40–50 h at 20°C. After centrifugation, each gradient tube was divided into 16 fractions using a gradient fractionator (Isco, USA) coupled with a syringe pump operated at 300 μl/min.

The density of the solution in each fraction was determined gravimetrically by weighing 100 μL aliquots and corrected from blank samples obtained from the same ultracentrifuge run. The RNA was then recovered from each gradient fraction by precipitation in 1 volume of ice-cold isopropanol (≥99.5%, Sigma Aldrich), incubation for 2 h at 4°C and centrifugation in a refrigerated microfuge (Eppendorf, Germany) at 14,000 x *g* for 30 min and 4°C. The pellets were resuspended in 30 μl MBG water, and stored at -20°C until further analysis.

### Real-time reverse transcription (RT) PCR for density gradient fractions

Total bacterial and archaeal biomass in gradient fractions was determined using real-time reverse transcription (RT)-PCR using the primer sets 1049F (5’-GTGSTGCAYGGYTGTCGTCA-3’) and 1194R (5’-ACGTCRTCCMCACCTTCCTC-3’) [28], and 340F (5’-CCCTAYGGGGYGCACAG-3’) and 1000R (5’-GGCCATGCACYWCYTCTC-3’) [29], respectively. The RT-qPCR reaction was carried out using 25 μL iScript One–Step RT-PCR SYBR Green mix (Biorad) and a C1000 Thermal cycler equipped with CFX96 Real-time system (Biorad) according to the reaction protocol (Biorad) and modified after Leuders et al. [30]. The thermal protocol for real-time PCR amplification was 120 s of initial denaturation (94°C), followed by 40 amplification cycles of 30 s at 94°C, 30 s at 52°C, and 60 s at 72°C. Bacterial and archaeal target copies were measured in experimental triplicates derived from the original triplicates (S1 Table). Standard curves were performed in triplicates using 16S rRNA gene clones of Dehalobacter restrictus (bacteria) and Methanococcoides burtonii (archaea).

### 16S rRNA gene pyrosequencing

Two gradient fractions (Fractions 11 –BD 1.785 g/ml and 4 –BD 1.838 g/ml) closest to the ‘light’ and ‘heavy’ RNA peaks respectively were selected for 16S amplicon pyrosequencing. For each fraction, 5 μl of RNA were reverse transcribed using AMV reverse transcriptase (Promega) according to the manufacturer’s instructions using the reverse primer 1392wR (5’-ACGGGCGGTGWGTRC-3’) at 20 μM final concentration. The reaction started at 65°C for 5 min, followed by reverse transcription at 37°C for 1 h and terminal elongation at 72°C for 10 min. The product cDNA was used as template for the production of 16S rRNA gene amplicons and amplified using the protocol described previously [21] using the same universal primers 926F (5’-AAACTYAAAKGAATTGACGG-3’) and 1392R (5’-ACGGGCGGTGTGTA-3’) modified on the 5’ end to contain 454 sequencing adaptor sequences [31].

Amplicons were pooled in equimolar concentrations and sequenced using a 454 GS-FLX Titanium platform (Roche Diagnostics, Castle Hill, Australia) as per the manufacturer’s instructions at the Australian Centre of Ecogenomics (ACE). Sequence data were processed and sequences with ≥97% similarity were assigned to operational taxonomic units (OTUs) using CD-HIT-OTU [32] (S2 Table). Each sequence was then classified using BlastTaxonAssigner in QIIME against the Greengenes database (http://greengenes.lbl.gov). Cluster identity at species level was assessed using phylogenetic analysis. The 16S rRNA sequence from the dominant clusters were aligned with existing sequences from the database using MEGA 5.10 (Molecular Evolutionary Genetics Analysis– www.megasoftware.net) imported into the ARB software.

## Results

### Biodegradation of acetate under high temperature anaerobic digestion

**Fig 1:**
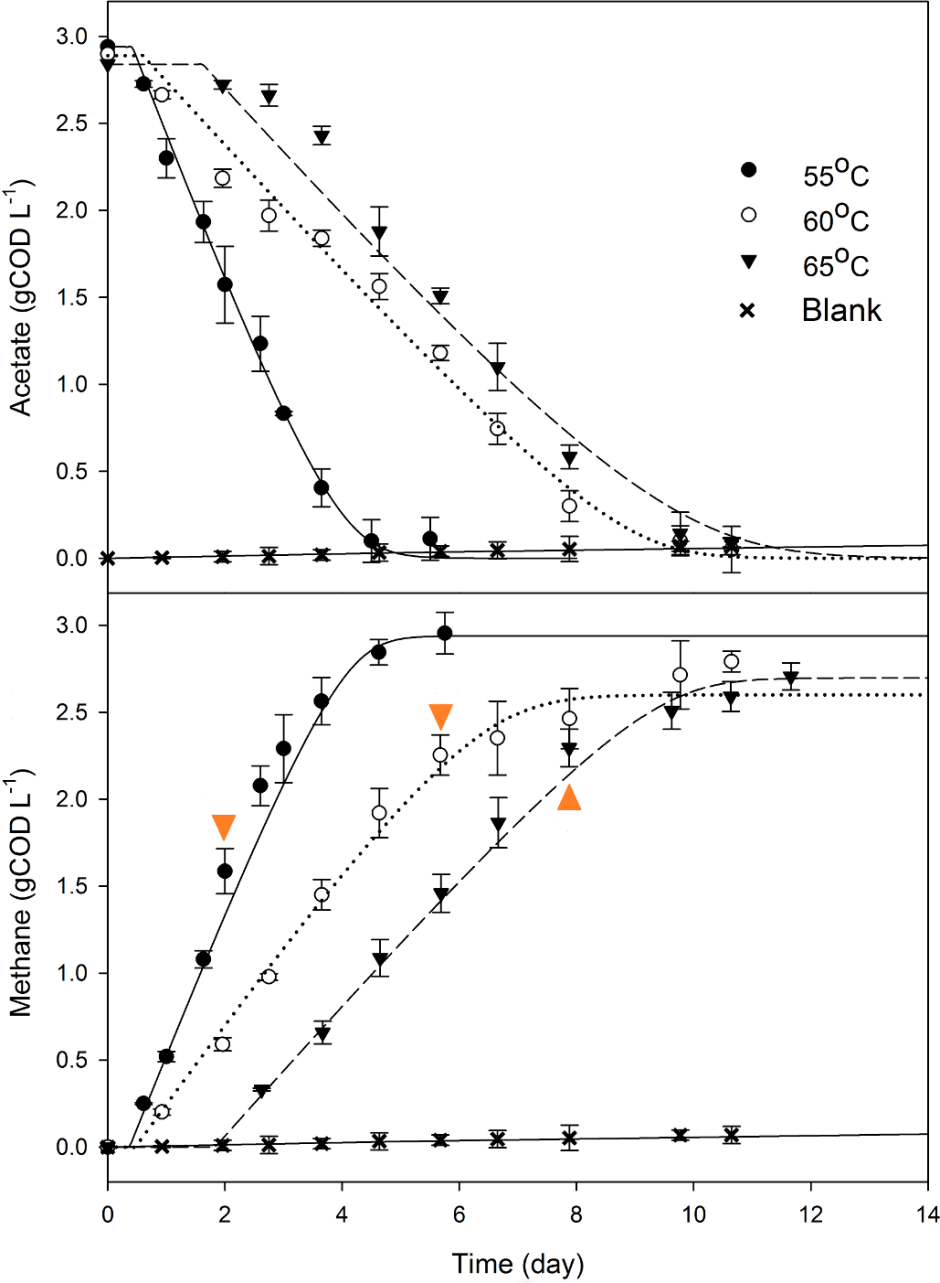
**The consumption of ^13^C-acetate (top) and the cumulative methane production (bottom) during the incubation at different temperatures.** The triangle symbol indicates when the biomass aliquots were collected for RNA extraction and isopycnic centrifugation, i.e. Day 2 for 55°C series, Day 6 for 60°C series, and Day 8 for 65°C series. Points refer to experimental data and lines to the optimised model. Error bars represent 95% CI in average of measurements from experimental triplicates (two-tailed *t-*test).

Acetate is the main precursor for methane production in engineered bioprocesses. Fig 1 presents the decrease in acetate concentrations and corresponding methane formation during the incubation of ^13^C-labelled acetate. The results for ^12^C-acetate (i.e, control) assays can be found in S3 Fig. Acetate uptake rates were estimated at 0.76 ± 0.027 g COD g VS^-1^ d^-1^ at 55°C and decreased to 0.30 ± 0.035 g COD g VS^-1^ d^-1^ at 60°C and 65°C (average measurement from experimental triplicates), which were consistent with rates determined previously [21]. The endogenic activities in all tests were negligible (blanks shown in Fig 1). A short lag phase of 1 to 2 days was observed at the higher incubation temperatures. Two-tailed *t-tests* identified there was no significant difference between the uptake rates for ^13^C-labelled and ^12^C unlabelled (control) acetate (*p*>0.05, S3 Table). To maximise ^13^C labelling while minimising cross-feeding, biomass was taken for molecular analysis after 60 to 80% of acetate consumption, which corresponded to Day 2 of the incubation at 55°C, Day 6 at 60°C, and Day 8 at 65°C.

### Quantification of bacterial and archaeal 16S rRNA

**Fig 2:**
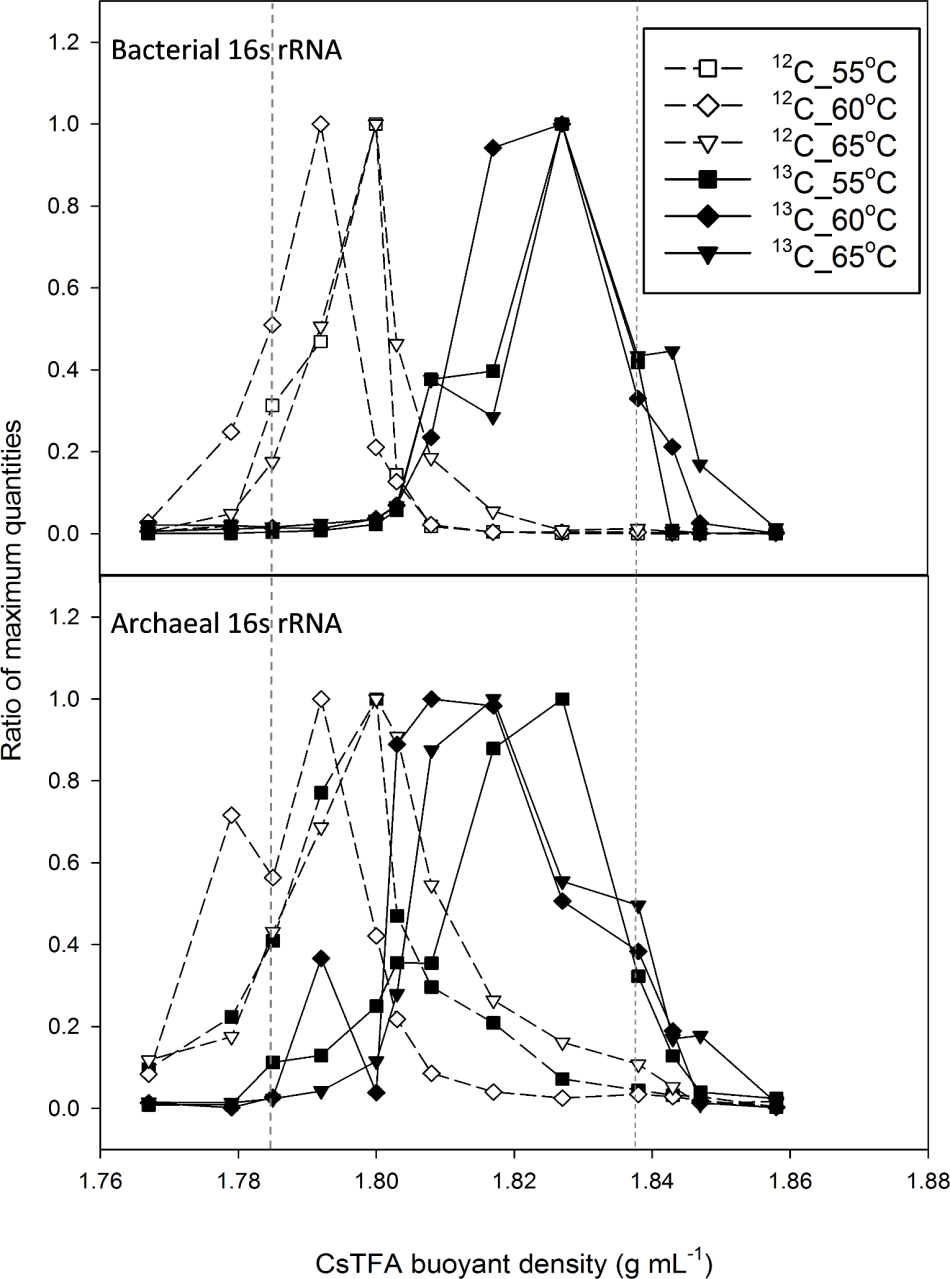
CsTFA buoyant density of rRNA extracted from unlabelled (^12^C) and labelled ^13^C-acetate incubations at 55 to 65°C. The RNA distribution amongst gradient fractions was quantified with domain-specific primers for bacteria and archaea using real-time RT-PCR. Fraction 11 (BD 1.785 g/ml) and Fraction 4 (BD 1.838 g/ml) from which 16S amplicon sequences were obtained and shown in Fig 2 are marked with vertical lines.

The abundances of bacterial and archaeal small subunit rRNA molecule copies in all gradient fractions (16 fractions per sample), as determined by RT-qPCR, varied from 10^6^ to 10^7^ and 10^4^ to 10^5^ ng of RNA per ml sample, respectively. The quantities varied slightly among the triplicates, probably due to the differences in the RNA concentrations lost during extraction, gradient processing and/or precipitation. The quantitative profiles of all samples are provided in S1 Table, and fraction vs BD for averages at each temperature shown in S4 Fig. The ratios of bacterial and archaeal rRNA gene copies in each gradient fraction relative to the maximum quantities collected in all fractions of one sample are presented in Fig 2.

The rRNA extracted from ^12^C-acetate treatments had RT-PCR peak buoyant densities (BD) between 1.77 and 1.81 g/ml CsTFA, which agrees with previous analysis of unlabelled rRNA [33]. In ^13^C-acetate treatments, bacterial 16S rRNA shifted towards heavy buoyant densities above 1.82 g/ml, which is indicative of strong ^13^C labelling of RNA. While archaeal rRNA buoyant densities were less differentiated between ^12^C and ^13^C substrate incubations with RNA from the labelled incubation showing a broader distribution of 1.80 to 1.84 g/ml, ^13^C labelling was evident at all temperatures (50 to 65°C). To identify primary acetate-degraders, two fractions were selected per sample for 16S amplicon sequencing including one ‘heavy’ fraction (Fraction 4, BD 1.838 g/ml) containing sequences from micro-organisms assimilating carbon from the ^13^C acetate and one ‘light’ fraction (Fraction 11, BD 1.785 g/ml) containing unlabelled RNA, as marked in Fig 2.

Bacterial peak location in ^13^C labelled samples was not substantially influenced by incubation temperature, but archaeal peak location shifted progressively to the heavier fraction as incubation temperature increased, indicating that they accumulated more label as temperature increased.

### Identification of acetate-utilisers

**Fig 3:**
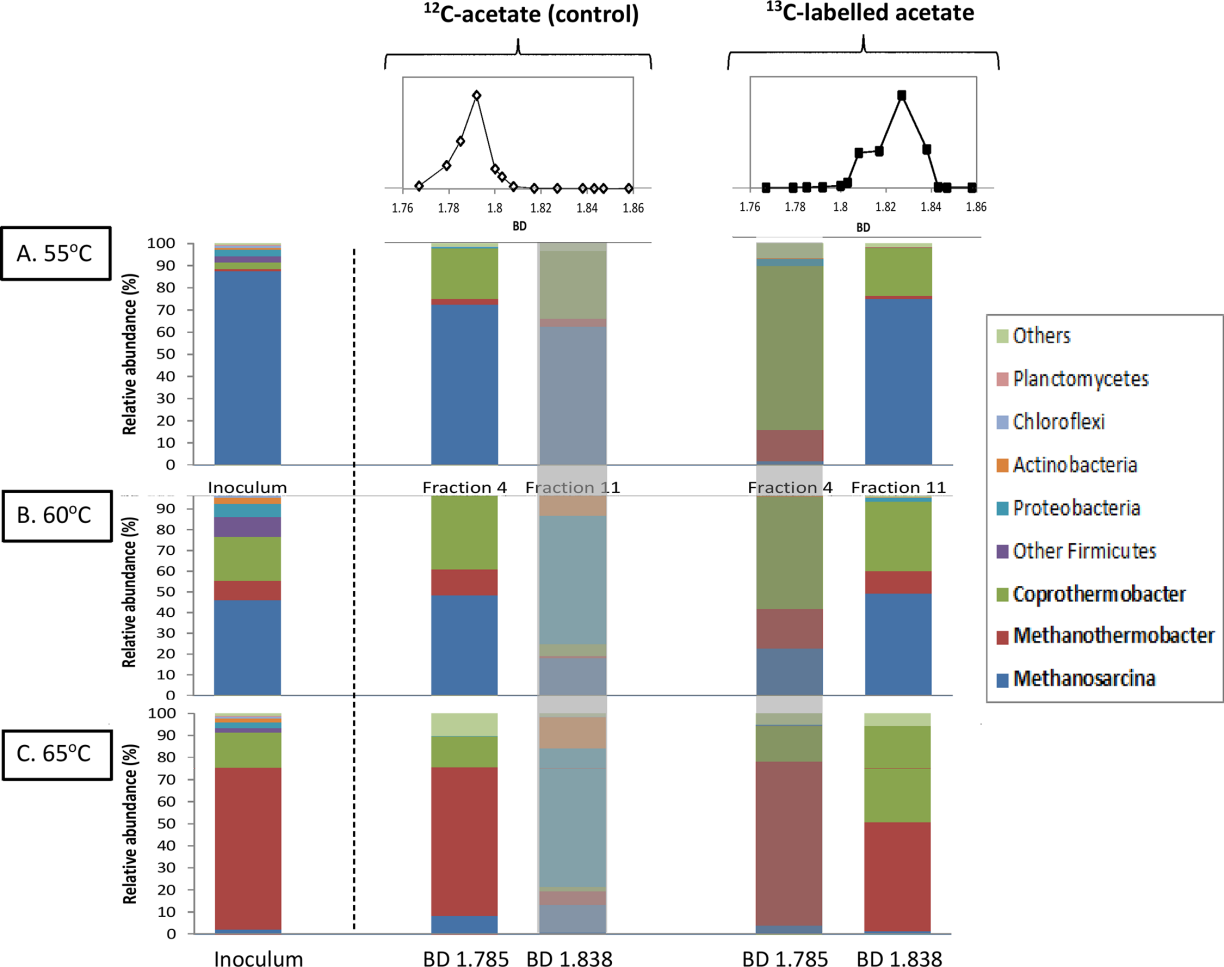
**Relative abundances of phylogenetic groups at 55°C (top group), 60°C (central group), and 65°C (bottom group).** From left to right: parent reactors (DNA), light RNA (BD 1.785) and heavy RNA (BD 1.838) fractions in ^13^C-acetate enriched, and ^12^C-acetate (control). Bacterial communities are presented by the dominating phyla while the archaeal *Euryachaeota* are presented by the dominating families of *Methanosarcinaceae* and *Methanobacteriaceae*. Phylogenetic groups accounting for ≤ 0.5% of all classified sequences are summarised in the artificial group ‘others’. Reference/residual fractions (light fraction for ^13^C experiment, heavy fraction for ^12^C control are greyed out).

Fig 3 presents the sequencing results including from left to right (a) parent community 16S pyrosequencing, community analysis, (b) the light (BD 1.785) and heavy (BD 1.838) fractions from ^13^C-labelled, and (c) the light and heavy fractions from the unlabelled assays. It is evident that in the presence of both labelled and unlabelled acetate, the dominant heavy and light fractions respectively were enriched, and largely match the dominant micro-organisms in the parent reactors (used as inoculum). Namely, *Coprothermobacter* appeared to be an abundant bacterial group at all temperatures, with methanogenic members shifting from *Methanosarcina* to *Methanothermobacter* progressively as temperature increased. While other members of phylum Firmicutes including potential syntrophic members of Clostridia were also present in the reactor communities (as shown in the inoculum profiles in Fig 3), they incorporation of ^13^C-labelled substrate in the ^13^C enriched gradient fractions (BD 1.838) was very low or not measured.

## Discussion

In this study, micro-organisms responsible for acetate degradation under thermophilic temperatures (55 to 65°C) were identified as two methanogenic genera of *Methanosarcina* and *Methanothermobacter* and syntrophic bacteria affiliated with the genus *Coprothermobacter*. Commonality between ^13^C-labelled (heavy RNA) and ^12^C-unlabelled (light RNA) indicates that the same metabolic processes were occurring with both ^13^C-acetate and ^12^C-acetate, and that labelled enrichment on ^13^C was matched by general enrichment on ^12^C due to growth on acetate. This is reasonable since the analysis was RNA based (i.e. focusing on active organisms), and the primary substrate in both cases was acetate. In addition, the light ^13^C-labelled (BD 1.785 g/ml) accumulated very small cell numbers (<2% of the maximum quantities), indicating that uptake of the label by key micro-organisms was very substantial.

### Competition and function of methanogens

The incorporation of ^13^C-acetate by *Methanosarcina* was anticipated due to its known role as an acetoclastic methanogen. The fraction of methane derived from acetate cleavage was 40% for the parent reactor at 55°C, previously quantified using stable carbon isotope fractionation [21]. The absence of *Methanosaeta* in the parent reactors was attributed to short operating SRT (3 days) and elevated temperatures (55 to 65°C), which selected only fast-growing thermophilic methanogens, and leaving *Methanosarcina* as the presumptive sole acetoclastic methanogen. No other H_2_ utilising methanogen was observed at 55°C (accumulating substrate), indicating *Methanosarcina* also acted as a hydrogen utiliser in syntrophy with *Coprothermobacter* as an acetate oxidiser.

As the incubation temperature increased the methanogenic population shifted from *Methanosarcina* to *Methanothermobacter*. The predominance of hydrogenotrophic methanogens in high-temperature anaerobic digesters were also reported elsewhere [13, 14] with SAO-HM accounting for a substantial fraction (40%) of acetate conversion at 55°C to almost all acetate conversion (>90%) at 65°C [21]. The loss of *Methanosarcina* corresponded to a decrease in acetate conversion rate and elevated residual acetate concentrations, as well as a shift towards SAO, and indicates that the presence of *Methanosarcina*, acting as AM, is a necessary condition to high-performance, high-temperature methanogenesis.

*Methanothermobacter* accumulating ^13^C-labelled carbon at 60°C and 65°C is likely a result of labelled bicarbonate transfer (cross-feeding) from *Coprothermobacter* (SAO-derived CH_4_ >90% [21]), since this methanogenic group has no known capability to utilise acetate as a carbon source [9]. Although cross-feeding is often regarded as a drawback of the RNA-SIP technique, it is actually important here to establish a syntrophy between hydrogenotrophic methanogens and acetate oxidiser(s) at the increased temperatures. Conducting time course analysis can overcome the limitation of single sample analysis in tracking the entry of labelled substrate into primary organisms and then either residing within them or passing to other organisms in the food-web over time [23, 34]. While this type of analysis is desirable it requires a much higher effort to ensure sufficient ^13^C labelling for RNA isolation and subsequent sequencing analysis.

Similarly, ^13^C labelling for secondary utilisers of ^13^C-acetate has been reported [34]. The detection of primarily hydrogenotrophic *Methanoculleus* spp. in high-density fractions from a DNA-SIP study was reported during the incubation of swine manure [34], this was an example of strong ^13^C-labelling for secondary utilisers and the predominance of SAO-HM methanogenesis in the system. However, no bacterial phylotypes could be detected in the ^13^C-DNA fractions in their study, highlighting the limitations of DNA-SIP when compared to RNA-SIP.

### Coprothermobacter as a potential acetate oxidiser

Members of genus *Coprothermobacter* accounted for a major part of the ^13^C-acetate assimilating populations at all temperatures. When considering i) very high conversion of acetate to methane and ii) the importance of SAO and HM in the continuous parent reactors [15], the results suggested that *Coprothermobacter* is the potential syntrophic acetate oxidiser. In a further attempt to characterise down to species level using neighbour joining method, the sequence was found closely related to *Coprothermobacter proteolyticous* (94% similarity, S5 Fig), previously isolated from an anaerobic digester operated at 55°C [35], and functionally linked to proteolytic degradation [36, 37]. Recently, Lu et al. [38] combined metaproteomics, isotopic analysis and pyrosequencing to investigate biological functions of thermophilic communities during cellulose anaerobic digestion. They retrieved specific enzymes (e.g. formyltetrahydrofolate synthetase proteins) associated with the oxidative acetyl-CoA pathway assigned to *C*. *proteolyticus*, suggesting their potential for performing SAO. Yet, direct involvement of this group in acetate degradation had not previously been proven.

*Coprothermobacter* is classified as part of the family Thermoanaerobacteriales. Previous culture studies have identified other potential candidates for SAO in this family including *Thermoacetogenium phaeum* isolated from a high temperature (55°C) anaerobic methanogenic reactor treating kraft-pulp wastewater [39], and *Tepidanaerobacter acetatoxydans* isolated from a lab-scale mesophilic bioreactor treating sludge containing high concentrations of ammonium [20]. The other mesophilic isolates belong to the physiological group of acetogens including *Clostridium ultunense* [18], and *Syntrophaceticus schinkii* [19]. Results here reveal another as-yet-uncultured acetate-oxidising candidate *Coprothermobacter* in the bioreactors operated at a temperature higher than 55°C and a short SRT of 3 days.

#### Implications for process design and operation

High-rate methanogenesis is uncommon at 37°C at SRTs of less than 10 days, but could be achieved when operating AD at high-temperature due to (i) enhanced methanogenic activities, (ii) the switch of acetotrophic metabolism to SAO-HM, and (iii) the maintenance of *Methanosarcina* with its AM ability and likely dual purpose of HM. The predominance of SAO over AM at high temperatures indicates higher endurance of the acetotrophic community against either temperature-induced inhibition or ammonia inhibition, and thus could be beneficial for the treatment of ammonia-rich wastes such as manure. In cases where methane production is targeted for optimisation a syntrophic acetate oxidiser, e.g. *Coprothermobacter* removes the bulk of acetate whereas *Methanosarcina* removes low level residuals, thus maintaining the quality of treated effluent. Increasing the digester temperature to >60°C drives the methanogenesis further to SAO-HM association with the exclusion of acetoclastic *Methanosarcina*, resulting in the accumulation of VFA. This higher temperature approach could be used to promote the production of both methane and higher value products (intermediate organic acids such as acetate, propionate, etc.) in a single reactor.

## Supporting Information

S1 Fig Methane production over time for the parent reactors operated at 55°C, 60°C, and 65°C Arrow indicates point at which inoculum was sampled. (TIF)

S2 Fig Volatile fatty acid (VFA) concentrations over time for the parent reactors operated at 55°C, 60°C, and 65°C. Arrow indicates point at which inoculum was sampled.
(TIF)

S3 Fig **The consumption of ^12^C-acetate (top) and the cumulative methane production (bottom) during the incubation at different temperatures.** The triangle symbol indicates when the biomass aliquots were collected for RNA extraction and isopycnic centrifugation, i.e. Day 2 for 55°C series, Day 6 for 60°C series, and Day 8 for 65°C series. Points refer to experimental data and lines to the simulation of the Monod model. Error bars represent 95% CI in average of measurements from technical triplicates (two-tailed *t-*test).
(TIF)

S4 Fig BD vs fraction numbers obtained as an average of each temperature. Global averages were used in Fig 2.
(TIF)

S5 Fig Phylogenetic tree based on the bacterial and archaeal 16S rRNA gene obtained from heavy fractions of SIP gradient and related type stain sequences using the neighbor-joining method in ARB. Scale bar, 10% estimated differences in nucleotide sequence. Numbers at each node are the percentage bootstrap value of 100 replicates. The characterised SAO organims including AF355615 (Thermotoga lettinga), GQ461825 (Clostridium ultunense), and AB020336 (Thermacetogenium phaeum) were used as the outgroup.
(TIF)

S1 Table Total archaeal and bacterial 16S rRNA gene copies in gradient fractions using real-time reverse transcription (RT)-PCR. (XLSX)

S2 Table OTU table obtained from QIIME v1.3.0 for the pyrosequencing of the parent reactors, light (BD 1.785) and heavy (BD 1.838) fractions of the ^12^C-control and ^13^C-labelled experiments (XLSX)

S3 Table Summary of p-values obtained from two-tailed student t-test for methane production rates compared across different temperatures between labelled and unlabelled (control) tests. (DOCX)
