# Supplementary figures and images for: High-rate, High Temperature Acetotrophic Methanogenesis Governed by a Three Population Consortium in Anaerobic Bioreactors
Source: PLoS One. 2016 Aug 4;11(8):e0159760. doi: 10.1371/journal.pone.0159760 (PMC4973872; doi:10.1371/journal.pone.0159760)

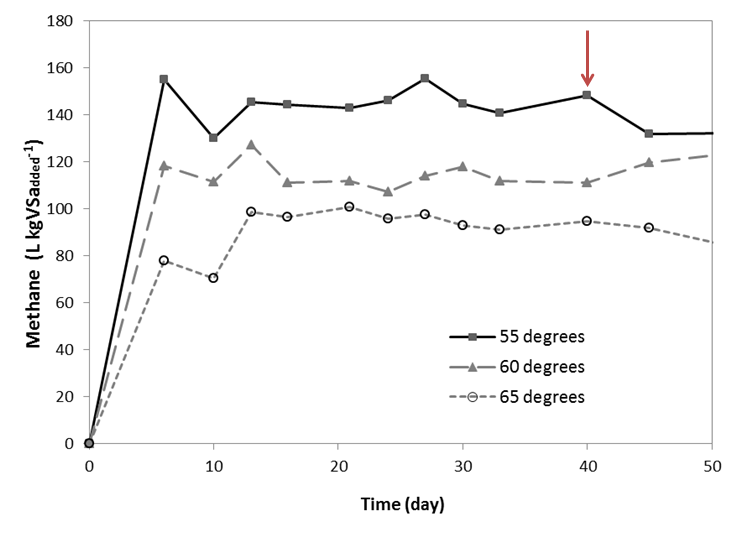

Supplement: S1 Fig — (TIF) [file pone.0159760.s001.tif]

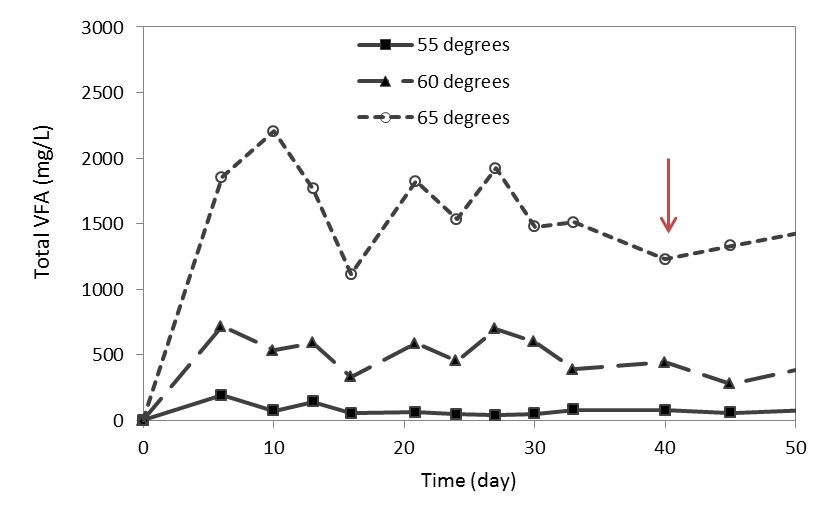

Supplement: S2 Fig — Arrow indicates point at which inoculum was sampled. (TIF) [file pone.0159760.s002.tif]

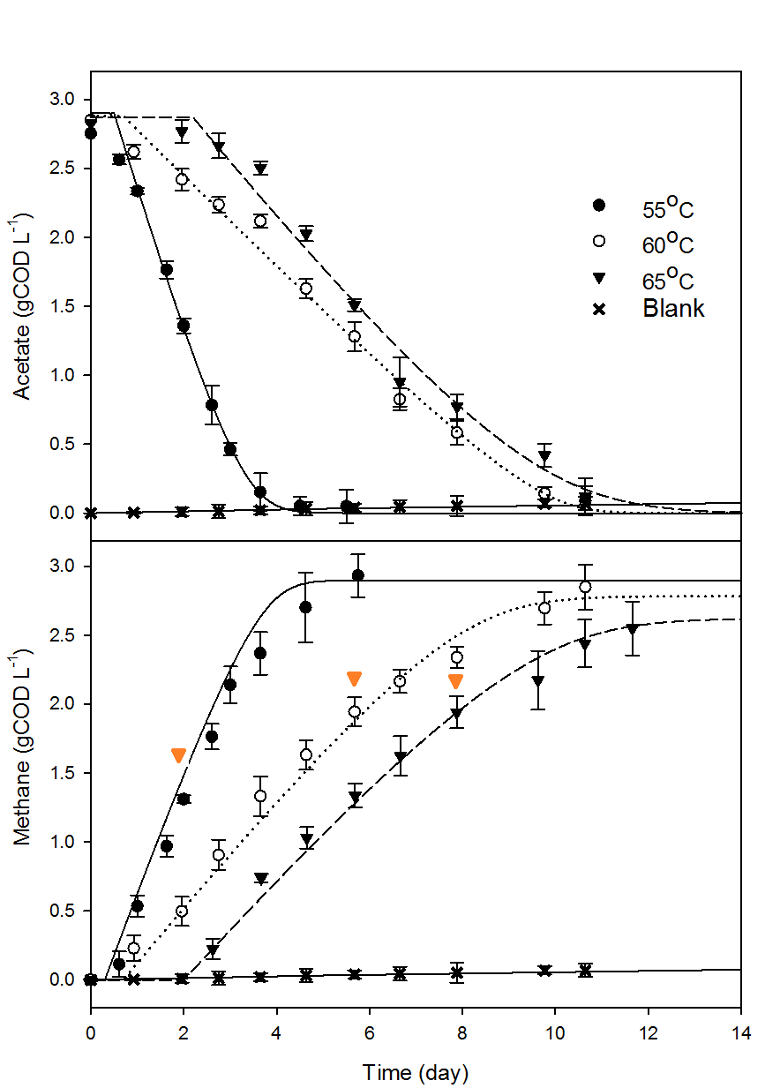

Supplement: S3 Fig — The consumption of 12C-acetate (top) and the cumulative methane production (bottom) during the incubation at different temperatures. The triangle symbol indicates when the biomass aliquots were collected for RNA extraction and isopycnic centrifugation, i.e. Day 2 for 55°C series, Day 6 for 60°C series, and Day 8 for 65°C series. Points refer to experimental data and lines to the simulation of the Monod model. Error bars represent 95% CI in average of measurements from technical triplicates (two-tailed t-test). (TIF) [file pone.0159760.s003.tif]

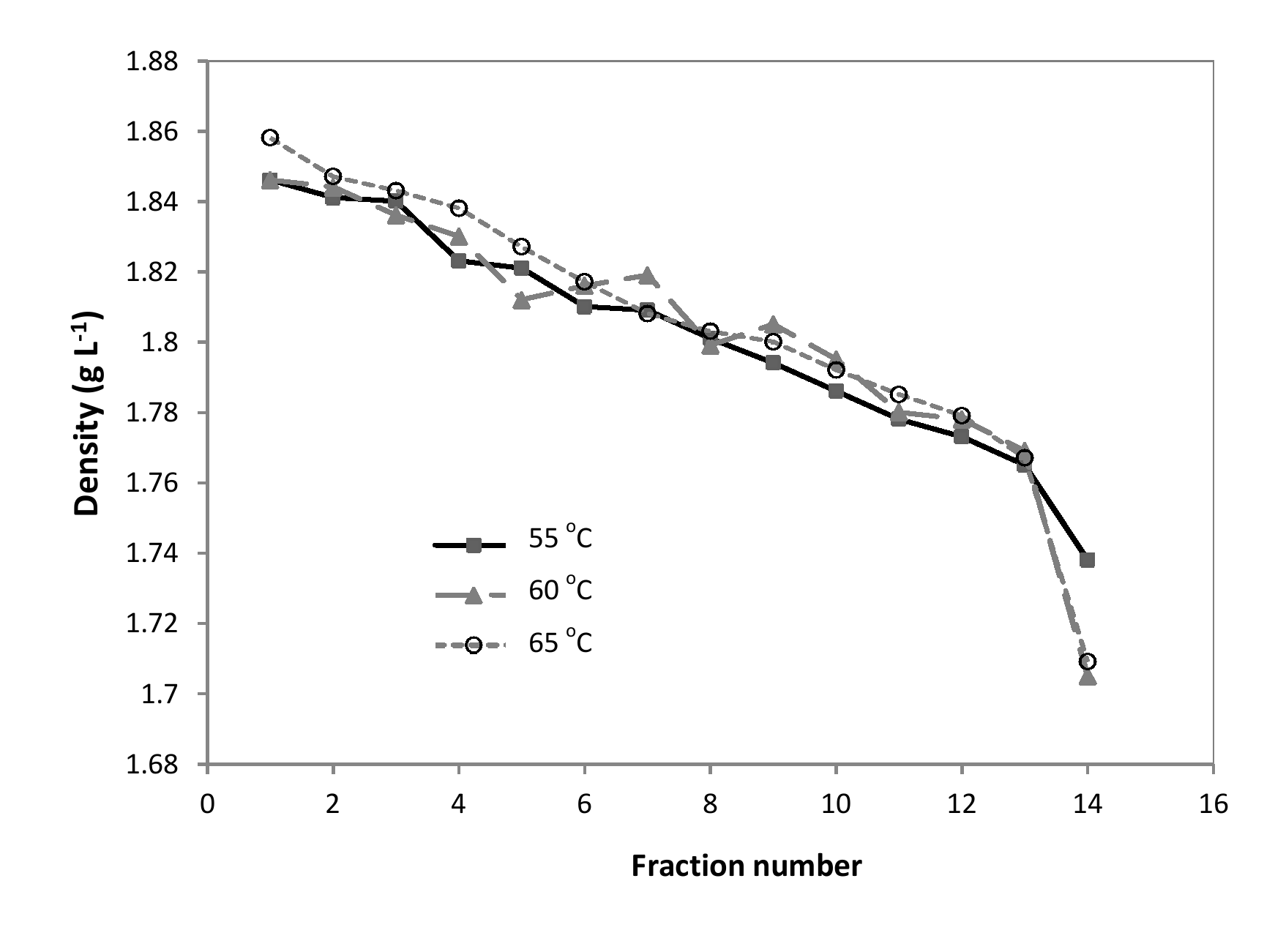

Supplement: S4 Fig — Global averages were used in Fig 2. (TIF) [file pone.0159760.s004.tif]

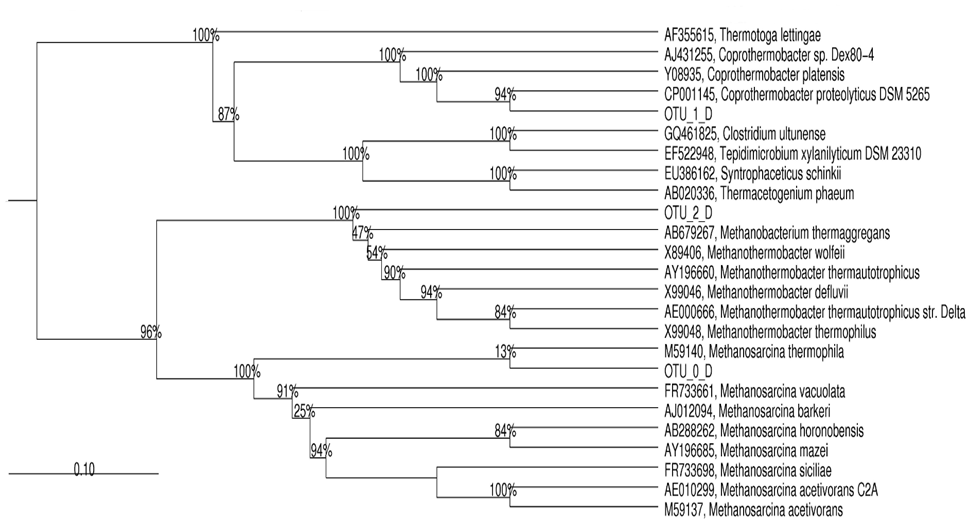

Supplement: S5 Fig — Scale bar, 10% estimated differences in nucleotide sequence. Numbers at each node are the percentage bootstrap value of 100 replicates. The characterised SAO organims including AF355615 (Thermotoga lettinga), GQ461825 (Clostridium ultunense), and AB020336 (Thermacetogenium phaeum) were used as the outgroup. (TIF) [file pone.0159760.s005.tif]
