# Supplementary material for: High-rate, High Temperature Acetotrophic Methanogenesis Governed by a Three Population Consortium in Anaerobic Bioreactors
Source: PLoS One. 2016 Aug 4;11(8):e0159760. doi: 10.1371/journal.pone.0159760 (PMC4973872; doi:10.1371/journal.pone.0159760)
Supplement: S3 Table — (DOCX) [file pone.0159760.s008.docx]

|  | p-value |
| --- | --- |
| 55^o^C | 0.77 |
| 60^o^C | 0.51 |
| 65^o^C | 0.50 |
